# Controversy on the CONVINCE study findings: the PRO take

**DOI:** 10.1590/2175-8239-JBN-2024-PO01en

**Published:** 2024-02-12

**Authors:** Bernard Canaud, Peter Blankestijn

**Affiliations:** 1 Montpellier University, Faculty of Medicine, Montpellier, France.; 2 MTX Consulting Int., Montpellier, France.; 3 University Medical Center Utrecht, Department of Nephrology and Hypertension, Utrecht, Netherlands.

**Keywords:** Kidney Failure, Chronic, Renal Replacement Therapy, Online Hemodiafiltration, Renal Dialysis, Patient Outcome Assessment, Falência Renal Crônica, Terapia de Substituição Renal, Hemodiafiltração On-Line, Diálise Renal, Avaliação de Resultados da Assistência ao Paciente

## Abstract

The CONVINCE study, recently published in the New England Journal of Medicine, reveals a groundbreaking 23% reduction in the relative risk of all-cause mortality among end-stage kidney patients undergoing high convective volume hemodiafiltration. This significant finding challenges the conventional use of high-flux hemodialysis and offers hope for improving outcomes in chronic kidney disease patients. While some controversies surround the study’s findings, including concerns about generalizability and the causes of death, it is essential to acknowledge the study’s design and its main outcomes. The CONVINCE study, part of the HORIZON 2020 project, enrolled 1360 patients and demonstrated the superiority of hemodiafiltration in reducing all-cause mortality overall, as well as in specific patient subgroups (elderly, short vintage, non-diabetic, and those without cardiac issues). Interestingly, it was shown that hemodiafiltration had a protective effect against infection, including COVID-19. Future research will address sustainability, dose scaling effects, identification of subgroups especially likely to benefit and cost-effectiveness. However, for now, the findings strongly support a broader adoption of hemodiafiltration in renal replacement therapy, marking a significant advancement in the field.

## RESUMO

O estudo CONVINCE, publicado recentemente no *New England Journal of Medicine*, revela uma redução inovadora de 23% no risco relativo de mortalidade por todas as causas entre pacientes renais em estágio terminal submetidos à hemodiafiltração de alto volume de convecção. Esse achado significativo desafia o uso convencional da hemodiálise de alto fluxo e oferece esperança de melhoria dos desfechos em pacientes com doença renal crônica. Embora algumas controvérsias cerquem os achados do estudo, incluindo preocupações sobre a generalização e as causas de óbito, é essencial reconhecer o desenho do estudo e seus principais desfechos. O estudo CONVINCE, parte do projeto HORIZON 2020, inscreveu 1.360 pacientes e demonstrou a superioridade da hemodiafiltração na redução da mortalidade por todas as causas em geral, bem como em subgrupos específicos de pacientes (idosos, HD de curta duração, não diabéticos e aqueles sem problemas cardíacos). Curiosamente, demonstrou-se que a hemodiafiltração teve um efeito protetor contra infecções, incluindo a COVID-19. Pesquisas futuras abordarão sustentabilidade, efeitos de escalonamento da dose, identificação de subgrupos especialmente propensos a se beneficiar e a relação custo-benefício. No entanto, por ora, os achados apoiam fortemente uma adoção mais ampla da hemodiafiltração na terapia renal substitutiva, marcando um avanço significativo na área.

## Introduction

The CONVINCE study was recently reported in the New England Journal of Medicine, showing a 23% reduction in the relative risk of death from all causes in the hemodiafiltration arm of patients receiving a high convective volume^1^. This is a groundbreaking finding that tends to prove that well-dosed hemodiafiltration is superior to high-flux hemodialysis in reducing the risk of mortality in end-stage kidney patients. This is already a tremendous achievement, considering that interventional studies in the advanced chronic kidney disease field that demonstrate a positive effect on primary outcomes are scarce or even non-existent in the recent decade^2^. Therefore, this result must be emphasized and certainly highlighted, as it opens new perspectives and brings hope to the renal replacement therapy field for chronic kidney disease patients, where outcomes remain relatively poor^3,4–5^.

Now, as already pointed out by some experts, there are ongoing arguments regarding the findings of the study^6,7^. These arguments contest the generalizability of the findings, raise further concerns regarding the causes of death (cardiovascular versus infection), and even put the burden of sustainability in the context of high-volume hemodiafiltration. Such controversial issues are a part of scientific life and must be acknowledged since they stimulate research and further analysis to understand the precise effects of hemodiafiltration on chronic kidney patients. However, these controversies should not diminish the value of the CONVINCE study’s design and its clinical implementation. It’s essential to consider also the context in which the study was developed, especially during the COVID-19 pandemic, and to acknowledge its main findings.

When analyzing the findings of an interventional randomized controlled study, one must address several questions in order to contextualize the study’s findings and draw the correct conclusions, as well as consider the practical implications in a clinical setting. This is what we will address in this controversial note in a straightforward manner.

## What is the Background of Convince?

The CONVINCE study was designed to settle a long-standing debate regarding the clinical benefits and superiority of hemodiafiltration compared to high-flux hemodialysis^8^. The story began with the initial findings of Euro-DOPPS in 2006, which demonstrated that hemodiafiltration, when administered with a convective volume above a certain threshold (20 liters) in postdilution mode, was associated with a significant reduction in mortality^9^. This finding generated significant interest within the scientific community, as it marked the first indication that a high convective dialysis dose could be beneficial for patient outcomes. Furthermore, this study indicated that hemodiafiltration (HDF) was associated with additional advantages over high-flux hemodialysis, such as reduced surrogate inflammatory markers and certain biomarkers. This finding motivated scientists to further explore the field^10^.

Following these findings, four randomized trials were launched in Europe: CONTRAST^11^, TURKISH HDF^12^, ESHOL^13^, and FRENCHY^14^. These trials aimed to explore the impact of HDF on mortality in comparison to hemodialysis. Among these trials, only one, namely ESHOL, successfully achieved the primary outcome by reducing all-cause mortality by 23% over a three-year follow-up period^13^. The other three studies did not show a significant benefit in terms of all-cause mortality, although there was a tendency toward reduced mortality in two of them^12,14^. Post-hoc analyses of these studies clearly identified that convective dose was the main differentiator between the studies and had an impact on patient outcomes. In this context, it was also demonstrated that the highest convective volumes, starting at 23 liters and above, were consistently associated with a reduction in mortality across all studies.

In light of these disappointing results, an initiative known as the “European HDF pooling project” was launched under the auspices of EUDIAL^15^, an ERA working group, to consolidate data from the four trials and conduct a new analysis^16,17^. To enhance the scientific value of this project, it was decided to proceed with an individual patient data meta-analysis (IPD-MA), following the approach described by the COCHRANE group. For this purpose, individual data from the four studies were collected up to the end of the patient follow-up period. The dataset was shared, reviewed, cleaned, and ultimately analyzed by an independent group of epidemiology experts from Oxford, focusing on clinical endpoints such as all-cause mortality and cardiovascular mortality. At the same time, sub-analyses of mortality were conducted by dividing convective volumes into tertiles and adjusting for various patient anthropometric characteristics, such as body weight, total body water, and body surface area. These analyses revealed a consistent trend: higher convective volumes were associated with better results. As highlighted in various reports from this IPD-MA study, there was a 14% reduction in overall mortality, with a significant 23% decrease in cardiovascular mortality. High volumes, specifically those in the upper tertile (set at 23 liters and above), consistently demonstrated a significant reduction in the risk of death, regardless of the adjustments made^16,17^. Cause-specific analysis revealed that all the benefits stemmed from cardiac-related causes, including congestive heart failure, ischemic events, and arrhythmias. Based on the IPD-MA, which enrolled over 2700 patients, it was established that a convective dose of 23 liters was the threshold required to reduce mortality in end-stage chronic kidney disease patients. This 23-liter convective dialytic dose served as the targeted dose for comparison in the CONVINCE study against high-flux hemodialysis^16,17^.

Interestingly, during this period, several observational studies were conducted. A new analysis by the DOPPS group during waves 4 and 5 (the later period) did not confirm the superiority of hemodiafiltration in this dataset^18^. However, the study had serious flaws, notably its failure to identify the substitution modality and convective dose delivered. In parallel, real-world evidence studies based on national registries (such as France: REIN^19^, Australia and New Zealand: ANZADATA^20^, and Japanese: JSDT^21^) were reported. These studies consistently confirmed a reduction in mortality ranging from 23% to 37% across all cases. Collectively, these studies suggest the superiority of high-volume hemodiafiltration in terms of outcomes compared to high-flux hemodialysis. Nevertheless, they leave clinical scientists with some uncertainty regarding the true value of these findings due to their potential bias.

## Why and How Convince was Designed?

To provide an indisputable answer to this remaining question—whether hemodiafiltration is superior to high-flux hemodialysis — the CONVINCE trial was proposed and designed, taking advantage of European funding through an innovative project, namely HORIZON 2020^22,23^. In line with HORIZON 2020 guidance, CONVINCE was structured to address two primary questions: the first one investigates hard clinical endpoints, such as mortality, while the second one assesses patient perception by focusing on health-related quality of life.

CONVINCE was designed as a pragmatic study, closely aligned with the standard practices of the various participating centers^1^. It did not introduce any additional burdens, such as specific laboratory tests or imaging measures. Patient management, dialysis prescriptions, and monitoring were conducted under the strict supervision of their respective nephrologists. The choice of dialysis machines and dialyzer brands and characteristics remained under the authority of the referring nephrologists.

The inclusion and exclusion criteria were traditional. Patients should have undergone hemodialysis for at least three months and should not have been previously exposed to hemodiafiltration. However, the most important selection criteria were, in fact, based on the perception of the referring nephrologists. During the patient screening process, referring nephrologists were asked to identify patients who met the study’s design requirements, specifically those who could achieve the targeted convective volume of 23 liters per session in postdilution hemodiafiltration.

## What Question was Convince Intended to Address?

As indicated above, CONVINCE was designed to address as primary endpoint all-cause mortality and as secondary endpoint cardiovascular events including mortality, but also infectious related complications and hospitalization^22^. Furthermore, patient-reported outcome measures (PROM) were specifically addressed with conventional tools including the health-related quality of life (HR-QOL SF36) questionnaire and also with development of a kidney disease-specific questionnaire as part of an innovative and computerized adaptive tool of the web-based PROMIS tool.

The role of the convective dose was crucial and considered the primary driver of the study when exploring hemodiafiltration. As previously indicated, a threshold of 23 liters of substitution volume was selected as the primary target, and its regular achievement was monitored throughout the 36-month follow-up period. As recently reported, this substitution volume was achieved in more than 90% of cases in almost all patients, with few deviations over time^24^. The total ultrafiltration volume delivered, which includes the substitution volume and net ultrafiltration volume required to restore fluid homeostasis, averaged around 26 liters per session. This total ultrafiltration volume should be considered as the effective convective dialytic dose delivered per session. Besides the target convective dose, all other key indicators of dialysis adequacy (fluid volume, blood pressure, anemia, mineral bone disease, nutritional status, etc.) should be maintained within the optimal range set by European best practice guidelines.

A particular focus of the CONVINCE study was on exploring the effect of hemodiafiltration compared to high-flux hemodialysis on patient-reported outcomes and health-related quality of life. This assessment was conducted using conventional tools as well as innovative self-adaptive computerized tools based on the PROMIS platform. For this purpose, a kidney disease-specific questionnaire was developed, validated, translated into various languages, and finally implemented with the support of a web-based electronic tablet tool. The results of patient perceptions have not been disclosed and are expected to be reported soon. This important and innovative aspect of the study could provide strong support for hemodiafiltration, if a positive impact is found.

## What are the Results?

Despite initial difficulties in implementation due to the COVID-19 pandemic outbreak, CONVINCE has finally enrolled 1360 end-stage kidney disease patients, making it the largest study comparing hemodiafiltration to high-flux hemodialysis today. As reported in the NEJM, hemodiafiltration reduces the relative risk of all-cause mortality by 23%. This confirms that the primary outcome was achieved and supports the hypothesis that adequately dosed hemodiafiltration is superior to high-flux hemodialysis, providing a more significant protective effect against mortality^1^.

In terms of secondary outcomes, there was an interesting trend suggesting a reduction in cardiovascular events, although no significant differences were observed in deaths from cardiovascular causes (HR 0.81 [0.49–1.33]) and/or other severe cardiovascular events (HR 1.07 [0.86–1.33]) between the hemodiafiltration and hemodialysis groups. Furthermore, another particularly intriguing finding was that the rate of death from infections, including COVID-19, was 21% lower in the hemodiafiltration group compared to the high-flux hemodialysis group. This observation might imply a more robust immune response to COVID-19 vaccine in patients undergoing hemodiafiltration, as reported in a recent study^25^. However, all these elements must be considered as working hypotheses, since one must consider that secondary outcomes should be approached with caution and in a more exploratory than explanatory manner, given that the final study was underpowered to address the COVID-19 outbreak and considering the initial statistical power plan.

In pre-specified subgroups, hemodiafiltration was shown to be superior for the elderly (>65 years old), non-diabetic patients, those with no history of cardiovascular issues, patients with arteriovenous fistulas, and those with a low dialysis vintage (<2 years).

## Do the Results Align with Our Expectations?

When comparing the findings of the CONVINCE study to those of the IPD-MA study, a few discrepancies may be noted^16,17^. Overall, the reduction in mortality risk falls within the same range as that observed in previous studies. However, despite a positive trend, cardiovascular risk reduction was not achieved in CONVINCE, whereas it was a primary finding in the European HDF Pooling project, with a mortality reduction of approximately 24%. This finding was further explored in a case-specific analysis of mortality among patients receiving HDF or HD^26^. This discrepancy raises a concern that requires further analysis for explanation. Additionally, it was observed that cardiac and diabetic patients derived greater benefits from hemodiafiltration in the previous European Pooling Project, a trend which has not been confirmed in CONVINCE.

Currently, the leading hypothesis to explain these discrepancies may be related to the COVID-19 outbreak, which significantly impacted the CONVINCE study. Most of the patients who died in the hospital during the COVID-19 pandemic were categorized as COVID-19-positive and death was not necessarily attributed to cardiovascular causes. In this context, it might be speculated that the cause of death was wrongly attributed to COVID-19 when in fact it had a cardiovascular cause. This hypothesis requires further analysis, which will involve revisiting medical records and may take some time. Furthermore, it could be conjectured that more fragile patients, such as those with diabetes or cardiac conditions, were more affected by COVID-19 infection. Supporting this hypothesis is the fact that hemodiafiltration has shown a protective effect and reduced mortality in other infected patients, including those who contracted COVID-19.

Whatever the exact explanations may be, they should not compromise the value of the original findings of the CONVINCE study.

## What are the Mechanisms that Support Clinical Benefits of Convince and Hemodiafiltration at Large?

This aspect has recently been comprehensively reviewed in several articles and will not be discussed here. We refer interested readers to previously published review articles^27,28^. In brief, one can recognize that the benefits of hemodiafiltration encompass a broad spectrum of biological and clinical effects, which can be categorized into two main categories: direct and indirect effects^28^.

A. Direct effects: These effects are related to the higher efficiency in removing medium and large uremic toxins, reducing inflammation and oxidative stress, improving the control of metabolic bone disease, enhancing hemodynamic stability with reduced dialysis-induced systemic stress, and reducing endothelial dysfunction.

B. Indirect effects: These effects are associated with improvements in nutritional parameters, increased physical activity, and enhanced correction of anemia with reduced erythropoietin consumption.

Collectively, these factors contribute to our current understanding of the improved clinical outcomes observed.

## Which Remaining Questions Need to be Addressed?

The CONVINCE study will continue to stand as a significant milestone in the field of end-stage kidney disease treatment. However, in keeping with the evolving nature of medical advancements, it will not represent the ultimate development of renal replacement therapy. Several lingering questions require immediate attention. In this regard, we can delve into three specific questions at this juncture.

The sustainability of hemodiafiltration in comparison to high-flux hemodialysis can be readily assessed with precise answers^7^. Currently, the medical devices and components employed in hemodiafiltration are virtually identical to those used in hemodialysis. Modern hemodialysis monitors can be considered a simplified version of hemodiafiltration machines since hemodiafiltration is an available option on all CE-marked dialysis machines equipped with sterilizing filters. Ultra-pure water quality is imperative for all types of hemodialysis treatments. Hemodialyzers and hemodiafilters share close similarities, differing mainly in a few internal geometric aspects, and standard high-flux hemodialyzers can be used as a default option^29^. In other words, this implies that the production of plastic waste will be similar to that of hemodialysis^30^. In the context of online hemodiafiltration and hemodialysis, the production and consumption of dialysis fluid are strictly identical and determined based on the user’s prescription. This aligns with the concept of online production, which ensures a perfect match between the dialysate entering and leaving the dialyzer, maintaining a precise balance of fluid volume^31^. Therefore, when the dialysis fluid production is set on a hemodiafiltration machine, a portion of the fresh dialysis fluid (typically 15–20% in postdilution mode) is diverted and infused directly into the bloodstream, while the deficit in volume at the outlet is compensated for by an increased ultrafiltration rate extracted from the patient’s blood. To illustrate, if the dialysis fluid production is set at 600 mL/min, mirroring the flow entering the dialyzer, the dialysate flow exiting the dialyzer at the outlet will also be 600 mL/min. Consequently, the consumption of dialysate flow remains consistent for both hemodialysis and hemodiafiltration sessions, reflecting the dialysate flow production. Additionally, innovative features of hemodiafiltration machines can align dialysate flow with blood flow to optimize this ratio^32^ and ensure automated ultrafiltration control to optimize ultrafiltration flow at any time^33^. From the perspective of solute clearance, this increase in ultrafiltration flow significantly enhances the clearance of medium and large molecular weight solutes, while it does not impact the clearance of small molecular weight solutes.

The generalizability and dose scaling effects are clearly concerns that need to be addressed for patients with different anthropometric profiles compared to European populations and/or various metabolic needs. The easiest way to tackle this problem is by adjusting the convective dialytic dose to the patient’s anthropometry. A simple nomogram or calculator could be employed to calculate the required convective dose, considering that the CONVINCE study has established a convective volume threshold of 14.5 liters per square meter of body surface area^16^. Following this straightforward guideline, it’s evident that lighter patients will require lower volumes while larger patients will necessitate higher volumes. For instance, the Asian population may require 20–22 liters, whereas the American population may require 26-28 liters per session. Another aspect to consider is the influence of blood flow and treatment time on convective dose delivery. In such cases, further specific local adjustments may be made. However, online hemodiafiltration should be regarded as a highly flexible tool for optimizing treatment for various patient profiles. Another aspect relates to the identification of patient profiles that can better benefit from HDF. This has been addressed in a recent study aimed at developing and validating a treatment effect prediction model to determine which patients would benefit the most from hemodiafiltration compared to hemodialysis in terms of all-cause mortality^34^. Notably, this sophisticated modelled approach found that patients who benefited the most from hemodiafiltration were younger, less likely to have diabetes or a cardiovascular history, and had higher serum creatinine and albumin levels. Interestingly, these patient characteristics closely align with the findings of the CONVINCE study.

Cost-effectiveness and cost-utility are currently under detailed investigation by a working group within the CONVINCE consortium. It is not our intention here to divulge specific findings, but it is reasonable to assume that any potential extra costs associated with hemodiafiltration would result from the increased life expectancy of patients, rather than additional features related to the treatment modality, as previously reported^35,36^. Furthermore, it should be noted that in most European countries, reimbursement for hemodiafiltration by healthcare payers is at a similar rate to that of high-flux hemodialysis. The only specific requirement for dialysis facilities offering hemodiafiltration is the obligation to conduct quarterly microbiological monitoring, including bacteriometry and endotoxin tests, of both water and dialysis fluid.

## How Findings of Convince Will Impact the Future of Renal Replacement Therapy?

The CONVINCE findings represent a significant advancement in the evolving paradigm of renal replacement therapy. As demonstrated by CONVINCE, the clinical implementation and safety of hemodiafiltration raised no major concerns in this extensive trial, which included various centers with diverse practices^24^. The observed reduction in mortality risk, a primary outcome in hemodiafiltration, translates into a substantial improvement in life expectancy for dialysis patients and enhances treatment tolerance.

As previously mentioned, further analyses will soon be conducted to solidify these findings or explore various other aspects, particularly patient perception, through extensive and innovative patient-reported outcome measurements that were performed. Additionally, hemodiafiltration is not the final and ultimate renal replacement modality. Further research is required, particularly in addressing the issue of protein-bound uremic toxins by enhancing adsorptive capacity or employing a combined approach with competitive binding substances.

## Take Home Message

Nonetheless, the CONVINCE findings represent another significant milestone in the field of renal replacement therapy, building upon the progress previously achieved with high-flux hemodialysis. To paraphrase Ralph Waldo Emerson, an American philosopher, hemodiafiltration is a journey and not a destination toward improving renal replacement therapy for chronic kidney disease patients. It is time to hemodiafiltration be widely adopted to improve the outcomes of kidney disease patients.
